# Supplementary material for: NAD+ Anabolism Disturbance Causes Glomerular Mesangial Cell Injury in Diabetic Nephropathy
Source: Int J Mol Sci. 2022 Mar 22;23(7):3458. doi: 10.3390/ijms23073458 (PMC8998683; doi:10.3390/ijms23073458)
Supplement: Supplementary file 1 [file ijms-23-03458-s001.zip › ijms-1627314-supplementary.pdf]

Supplementary Materials

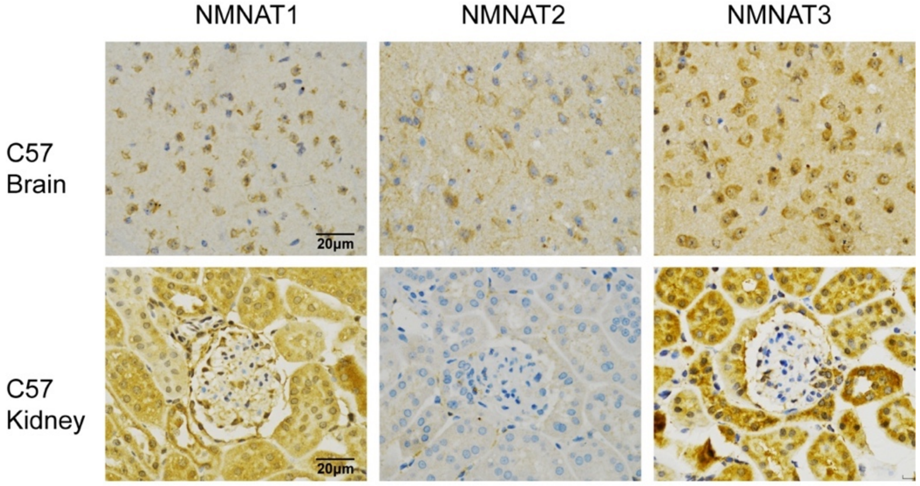

Supplementary 1. The expression of NMNATs in mice kidney and brain. Immunohistochemical staining was used to detect the expression and localization of NMNATs in brain and kidney of male C57BL/6J mice. 20×10 magnification by light microscope. The scale bar=20μm.

The sequence of NMNAT1:

GATTACAAGGATGACGACGATAAGGAAAATTCCGAGAAGACTGAAGTGGTTCTCCTTGCTTGT
GGTTCATTCAATCCCATCACCAACATGCACCTCAGGTTGTTTGAGCTGGCCAAGGACTACATGA
ATGGAACAGGAAGGTACACAGTTGTCAAAGGCATCATCTCTCCTGTTGGTGATGCCTACAAGA
AGAAAGGACTCATTCCTGCCTATCACCGGGTCATCATGGCAGAACTTGCTACCAAGAATTCTAA
ATGGGTGGAAGTTGATACATGGGAAAGTCTTCAGAAGGAGTGGAAAGAGACTCTGAAGGTGCT
AAGACACCATCAAGAGAAATTGGAGGCTAGTGACTGTGATCACCAGCAGAACTCACCTACTCT
AGAAAGGCCTGGAAGGAAGAGGAAGTGGACTGAAACACAAGATTCTAGTCAAAAGAAATCCC
TAGAGCCAAAAACAAAAGCTGTGCCAAAGGTCAAGCTGCTGTGTGGGGCAGATTTATTGGAGT
CCTTTGCTGTTCCCAATTTGTGGAAGAGTGAAGACATCACCCAAATCGTGGCCAACTATGGGCT
CATATGTGTTACTCGGGCTGGAAATGATGCTCAGAAGTTTATCTATGAATCGGATGTGCTGTGG
AAACACCGGAGCAACATTCACGTGGTGAATGAATGGATCGCTAATGACATCTCATCCACAAAA
ATCCGGAGAGCCCTCAGAAGGGGCCAGAGCATTCGCTACTTGGTACCAGATCTTGTCCAAGAA
TACATTGAAAAGCATAATTTGTACAGCTCTGAGAGTGAAGACAGGAATGCTGGGGTCATCCTG
GCCCCTTTGCAGAGAAACACTGCAGAAGCTAAGACATAG

The sequence of NAMPT:

GATTACAAGGATGACGACGATAAGAATCCTGCGGCAGAAGCCGAGTTCAACATCCTCCTGGCC
ACCGACTCCTACAAGGTTACTCACTATAAACAATATCCACCCAACACAAGCAAAGTTTATTCCT
ACTTTGAATGCCGTGAAAAGAAGACAGAAAACTCCAAATTAAGGAAGGTGAAATATGAGGAA
ACAGTATTTTATGGGTTGCAGTACATTCTTAATAAGTACTTAAAAGGTAAAGTAGTAACCAAAG
AGAAAATCCAGGAAGCCAAAGATGTCTACAAAGAACATTTCCAAGATGATGTCTTTAATGAAA
AGGGATGGAACTACATTCTTGAGAAGTATGATGGGCATCTTCCAATAGAAATAAAAGCTGTTCC
TGAGGGCTTTGTCATTCCCAGAGGAAATGTTCTCTTCACGGTGGAAAACACAGATCCAGAGTGT
TACTGGCTTACAAATTGGATTGAGACTATTCTTGTTCAGTCCTGGTATCCAATCACAGTGGCCAC

AAATTCTAGAGAGCAGAAGAAAATATTGGCCAAATATTTGTTAGAAACTTCTGGTAACTTAGAT
GGTCTGGAATACAAGTTACATGATTTTGGCTACAGAGGAGTCTCTTCCCAAGAGACTGCTGGCA
TAGGAGCATCTGCTCACTTGGTTAACTTCAAAGGAACAGATACAGTAGCAGGACTTGCTCTAAT
TAAAAAATATTATGGAACGAAAGATCCTGTTCCAGGCTATTCTGTTCCAGCAGCAGAACACAGT
ACCATAACAGCTTGGGGGAAAGACCATGAAAAAGATGCTTTTGAACATATTGTAACACAGTTTT
CATCAGTGCCTGTATCTGTGGTCAGCGATAGCTATGACATTTATAATGCGTGTGAGAAAATATG
GGGTGAAGATCTAAGACATTTAATAGTATCAAGAAGTACACAGGCACCACTAATAATCAGACC
TGATTCTGGAAACCCTCTTGACACTGTGTTAAAGGTTTTGGAGATTTTAGGTAAGAAGTTTCCTG
TTACTGAGAACTCAAAGGGTTACAAGTTGCTGCCACCTTATCTTAGAGTTATTCAAGGGGATGG
AGTAGATATTAATACCTTACAAGAGATTGTAGAAGGCATGAAACAAAAAATGTGGAGTATTGA
AAATATTGCCTTCGGTTCTGGTGGAGGTTTGCTACAGAAGTTGACAAGAGATCTCTTGAATTGTT
CCTTCAAGTGTAGCTATGTTGTAACTAATGGCCTTGGGATTAACGTCTTCAAGGACCCAGTTGCT
GATCCCAACAAAAGGTCCAAAAAGGGCCGATTATCTTTACATAGGACGCCAGCAGGGAATTTT
GTTACACTGGAGGAAGGAAAAGGAGACCTTGAGGAATATGGTCAGGATCTTCTCCATACTGTCT
TCAAGAATGGCAAGGTGACAAAAAGCTATTCATTTGATGAAATAAGAAAAAATGCACAGCTGA
ATATTGAACTGGAAGCAGCACATCATTAG

All cDNAs were synthesized and detected by a sequencing company (GENEWIZ, Suzhou, China)
